# Mortality Trends in Patients Undergoing Hemodialysis, 2003–2021: Data from National Health Insurance Service in Korea

**DOI:** 10.3390/jcm14092987

**Published:** 2025-04-25

**Authors:** Kyung Won Kim, Yoonjong Bae, Jee Young Lee, Young-Il Jo, AJin Cho

**Affiliations:** 1 Division of Nephrology, Department of Internal Medicine, Konkuk University Medical Center, Konkuk University School of Medicine, Seoul 05030, Republic of Korea; 20240544@kuh.ac.kr (K.W.K.); 20220478@kuh.ac.kr (J.Y.L.); nephjo@kuh.ac.kr (Y.-I.J.); 2 Department of Data Science, Hanmi Pharm. Co., Ltd., Seoul 05545, Republic of Korea; yoonjong.bae@hanmi.co.kr

**Keywords:** mortality, hemodialysis, end-stage kidney disease, chronic kidney disease, trend analysis

## Abstract

**Background**: Assessing recent changes in mortality among patients undergoing hemodialysis (HD) can help both to identify the causes of death most closely associated with these changes and to develop prevention strategies. This study explored trends in all-cause and cause-specific mortality among patients undergoing HD in South Korea using an analysis of national data. **Methods:** We used national death certificate and claims data from 2003 to 2021 provided by the National Health Insurance Service. Age-standardized mortality rates (ASRs) were calculated by standardizing to the 2011 population of patients undergoing HD. Joinpoint regression analysis was performed to calculate the annual percentage change (APC) in mortality. All-cause and cause-specific ASRs and APCs were evaluated for the study period. **Results**: The proportion of male and older adult patients increased over time. In particular, the proportion of patients aged ≥ 80 years in the 2018–2021 period was more than 4 times higher than in the 2003–2007 period. From 2003 to 2021, there were a total of 136,302 deaths among patients undergoing HD in South Korea. Cardiovascular causes accounted for 13.6% of deaths, and the majority (86.4%) were attributed to noncardiovascular causes. In 2003, the all-cause ASR was 174.1 per 1000 person-years, which steadily decreased to 114.5 per 1000 person-years in 2021. The ASR from cardiovascular disease remained unchanged from 2003 to 2013 but increased by 3.9% (95% confidence interval: 1.3 to 14.0) per year from 2013 to 2021. In contrast, the ASR from noncardiovascular disease decreased during the study period. **Conclusions:** Nationally representative data showed a declining trend in the ASR among patients undergoing HD from 2003 to 2021. Noncardiovascular disease mortality decreased during the study period, while cardiovascular disease mortality increased.

## 1. Introduction

Over the past two decades, significant technological and quality advances in hemodialysis (HD) procedures have been made [1,2]. Several large-scale studies have shown that advances in clinical care, new drug development, and improved management of comorbidities and malnutrition have led to modest improvements in survival among patients undergoing HD. The United States Renal Data System (USRDS) shows a decrease in all-cause mortality for patients who underwent HD between 1977 and 2007 [3]. According to analyses of data from the Japanese Society for Dialysis Therapy Renal Data Registry (JRDR), mortality rates between 1988 and 2013 decreased markedly among Japanese patients undergoing HD [4].

However, patients undergoing HD still have a higher risk of death compared with the general population [5,6]. Evaluating mortality trends among patients undergoing HD is necessary to account for past and present serious health risks. Cardiovascular disease is the most common cause of death in these patients, which was recognized more than 40 years ago [7]. Several studies have shown that cardiovascular disease accounts for 40–50% of deaths in patients with end-stage kidney disease (ESKD) [8,9,10]. Thus, evaluating mortality trends in patients undergoing HD is necessary to account for past and present serious health risks. However, though cardiovascular disease has been the leading cause of death in patients undergoing HD, recent advances in cardiovascular disease treatment have likely changed this trend. The JRDR data showed that the risk of cardiovascular death has decreased, while the risk of death from infection has remained unchanged for 25 years in patients undergoing HD [4]. Few recent reports have assessed trends in cardiovascular and noncardiovascular mortality in these patients. Understanding recent changes in cause-specific mortality among patients undergoing HD can help identify the causes of death most closely associated with changes in mortality and develop prevention strategies. Thus, the present study examined all-cause and cause-specific mortality trends in South Korean national-level data from 2003 to 2021 among patients undergoing HD.

## 2. Methods

### 2.1. Data Sources

We used the 2003–2021 National Health Insurance Service (NHIS) death certificate and claim data. These data include information regarding reimbursed medical services, which contain details about diseases and inpatient and outpatient usage records classified by diagnosis. In South Korea, the NHIS provides mandatory insurance, with 97% of the population enrolled in its National Health Insurance program. The study was conducted in accordance with the Declaration of Helsinki. The database was fully anonymized, and the requirement of informed consent was waived by the Ethics Committee of the Institutional Review Board of Hallym University Kangnam Sacred Heart Hospital (IRB No. HKS 2023-01-021).

### 2.2. Study Population

We selected the ESKD cohort patient data from the NHIS database based on the International Classification of Diseases, Tenth Revision (ICD-10) codes and intervention procedures. We initially identified patients who were diagnosed with chronic kidney disease between 1 January 2003 and 31 December 2021, based on the presence of ICD-10 codes (i.e., N18, N18.1–18.6, N18.9). Patients with ESKD were selected using special exemption codes (V001: HD; V003: peritoneal dialysis; and V005: kidney transplant) provided by the South Korean government. The South Korean government provides financial support through the NHIS to patients with rare, incurable diseases such as ESKD. When such diagnoses are confirmed by physicians and registered with the NHIS, they receive a special exemption code for a reduction in coinsurance rates and medical expenses of up to 90%. Therefore, most patients with ESKD who start HD in Korea are covered by the National Health Insurance and are granted a rare disease exemption code. Patients undergoing HD were classified based on these special exemption codes and procedure codes from NHIS claims. Target patients were those with ESKD who had received HD for more than 3 months and were aged > 20 years. We included both patients who started HD during the study period and those who had begun HD prior to the study period.

### 2.3. Outcome Measurement

Study outcomes were all-cause and cause-specific mortality. The cause of death was defined according to the ICD-10 coding system (Table S1). We defined cardiovascular mortality as deaths attributed to heart failure or cardiomyopathy, ischemic heart disease, valvular heart disease, arrhythmia, hyperkalemia/sudden death, cerebrovascular disease, and pulmonary embolism. Noncardiovascular mortality was defined as death attributed to causes other than cardiovascular causes. Cause-specific mortality included heart failure or cardiomyopathy, ischemic heart disease, hyperkalemia/sudden death, cerebrovascular disease, infection, and malignancy. Comorbidities for patients who started HD were determined by referring to claims data from one year prior to the HD start date, with the corresponding ICD-10 codes (Table S2) used at least once during an inpatient stay and at least twice during an outpatient stay.

### 2.4. Statistical Analyses

We calculated age-standardized mortality rates (ASRs) per 1000 person-years. Unadjusted age-specific rates were calculated by dividing the number of deaths by the cumulative number of person-years for each age group, age group of 5-year interval. We estimated the number of person-years as the median of the population for each year. We then calculated age-standardized mortality rates (ASRs) using the direct method. We used the 2011 population of patients undergoing HD as our standard population. The results were stratified and analyzed according to sex, cardiovascular/noncardiovascular cause of death, and specific causes of death.

We used joinpoint regression analysis to calculate the average annual percentage change (APC) in ASRs. In this analysis, we determined the points where the linear slope of the mortality trend changed significantly over the analysis period. It was assumed that the ASRs followed a Poisson distribution. *p* < 0.05 was considered a statistically significant change. The APCs of ASRs for each period and their corresponding 95% confidence intervals (CIs) were estimated and then tested to determine whether the data deviated significantly from the null hypothesis. For mortality rates in the general population, we used data from the National Center for Statistics [11]. Statistical analyses were performed with SAS software (version 9.4; SAS Institute, Cary, NC, USA).

## 3. Results

### 3.1. Study Population

Table 1 shows the crude mortality rate for the study population and the general population. While in the general population, mortality rates for all age groups have decreased over time, patients undergoing HD show a different trend. Patients undergoing HD show a decreasing trend in mortality, mainly in the 60 and over age group. Table 2 shows the age and gender distribution of the study population. The proportion of male and older adult (aged ≥ 65 years) patients increased over time. In particular, the proportion of those aged ≥ 80 years in the 2018–2021 period was more than 4 times higher than in the 2003–2007 period. Patients who started HD during the study period were predominantly male, and there was a continuous increase in the proportion of older adults (Table S3). The number of patients with comorbidities at the start of HD, including diabetes, hypertension, and heart failure, increased over time.

### 3.2. Mortality Trend

From 2003 to 2021, a total of 136,302 deaths occurred among patients undergoing HD in South Korea. Among these, 13.6% and 86.4% were due to cardiovascular and noncardiovascular diseases, respectively. Specifically, heart failure or cardiomyopathy, ischemic heart disease, cerebrovascular disease, hyperkalemia/sudden death, infectious diseases, and malignancy accounted for 1.5%, 5.0%, 4.9%, 1.1%, 5.9%, and 10%, respectively, of all deaths (Table S4).

**Figure 1:**
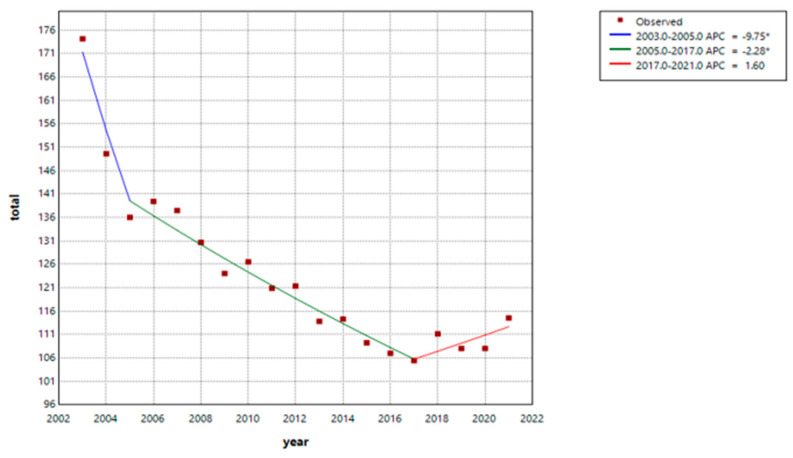
Age-standardized rates for all-cause mortality, 2003–2021. * indicates that the Annual Percent Change (APC) is significantly different from zero at the alpha = 0.05 level. The line is based on joinpoint analysis.

All-cause ASR in 2003 was 174.1 per 1000 person-years and declined steadily thereafter to 114.5 per 1000 person-years in 2021 (Table 3). Overall, the all-cause ASR during the ~20-year study period showed a steady decrease among patients undergoing HD (Figure 1). All-cause ASR decreased continuously by −9.8% (95% CI: −12.3 to −3.3) per year from 2003 to 2005 and then by −2.3% (95% CI: −3.6 to −1.1) per year from 2005 to 2017 (Table 4). The ASR did not change from 2017 to 2021. All-cause mortality trends were similar for female and male patients (Table 2, Tables S5 and S6).

**Figure 2:**
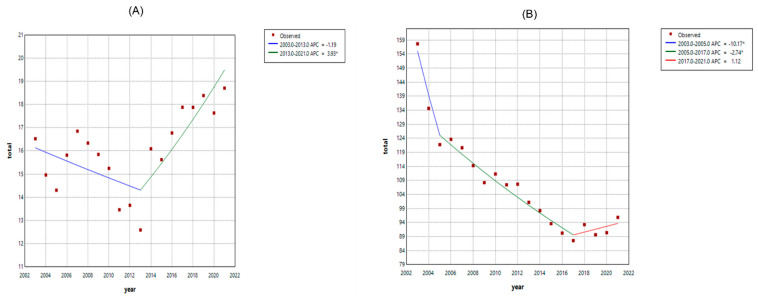
Age-standardized rates for cardiovascular (**A**) and noncardiovascular (**B**), 2003–2021. * indicates that the Annual Percent Change (APC) is significantly different from zero at the alpha = 0.05 level. Lines are based on joinpoint analysis.

Figure 2 shows mortality trends from cardiovascular and noncardiovascular causes. The ASR from cardiovascular disease did not change significantly from 2003 to 2013 but increased by 3.93% (95% CI: 1.3 to 14.0) per year from 2013 to 2021. Meanwhile, the ASR from noncardiovascular disease decreased by −10.2% (95% CI: −12.7 to −4.0) per year from 2003 to 2005 and then by −2.7% (95% CI: −3.8 to −1.7) per year from 2005 to 2017 (Table 4).

### 3.3. Trends in Mortality Rates by Cause

The ASR due to ischemic heart disease decreased from 2003 to 2021, with a significant decrease of −1.8% (95% CI: −2.9 to −0.6) per year, especially for women (Supplementary Materials). ASR due to heart failure or cardiomyopathy increased by 30.1% (95% CI: 17.7 to 70.7) per year from 2003 to 2009 and by 15.6% (95% CI: 7.3 to 50.5) per year from 2012 to 2021. The ASRs from heart failure or cardiomyopathy for women and men increased by 11.8% (95% CI: 9.1 to 14.5) and 11.4% (95% CI: 4.8 to 18.4) per year, respectively, from 2003 to 2021 (Supplementary Materials). The ASR for cerebrovascular diseases decreased from 2003 to 2012, after which it remained unchanged. The mortality rate for hyperkalemia decreased from 2003 to 2006, then increased until 2009, after which it did not change significantly. The rate of mortality from infection showed a mixed trend; however, the trend in women has increased from 2013 to 2021 (Supplementary Materials). Cancer-related mortality rates showed a decreasing trend. The ASR due to cancer decreased by 17.4% (95% CI: −23.7 to −13.3) per year from 2003 to 2007 and by −0.9% (95% CI: −1.7 to 0.005) per year from 2007 to 2021.

## 4. Discussion

In this analysis of nationwide South Korean death certificate data, we identified decreasing trends in mortality from 2003 to 2021, with patterns varying by specific cause. In terms of overall mortality reduction, the results are similar to those reported in previous studies, which have shown a significant decline over the last 20–40 years [3,12]. An earlier study of survival trends among patients with ESKD who were receiving long-term HD in the United States found that age-specific survival trends improved from 1977 to 2007. Despite the increasing age of patients with ESKD, the authors of the study observed a decrease in life expectancy loss from 23.6 years in 1977 to 19.7 years in 2007 among patients undergoing HD [3]. According to a recent USRDS report, patients undergoing HD showed decreased adjusted mortality from 179.4 per 1000 person-years in 2012 to 166.0 per 1000 person-years in 2019 [13]. In the present study, the all-cause ASR was lower than in the USRDS report, likely due to differences in health-care settings and insurance status between these countries.

Analysis of data from the European Renal Association–European Dialysis and Transplant Association Registry from 1998 to 2011 showed a decreased risk of cardiovascular death over the past decade [14]. An analysis of the 1992–2005 Australian and New Zealand Dialysis and Transplantation Registry found that cardiovascular disease mortality decreased among patients undergoing HD who were aged ≥ 55 years, while the relative risk of cardiovascular disease in patients with ESKD increased over time compared with the general population [5]. Storey et al. also reported that noncardiovascular mortality had declined more rapidly in patients with ESKD compared with controls, but that cardiovascular mortality declined more slowly [12]. This can be explained by the significant proportion of cardiovascular mortality in patients with ESKD being associated with nonatherosclerotic cardiovascular disease, which is less susceptible to changes in traditional risk factors because of its complex etiology [15,16]. Meanwhile, the decline in cardiovascular disease mortality in the general population is largely the result of public health improvements aimed at reducing cardiovascular disease risk factors [17].

Despite these differences in treatment and cardiovascular disease risk factors between patients undergoing HD and the general population, the absolute risk of cardiovascular death in older patients undergoing HD may have decreased over time, suggesting that some aspects of management have improved. In a study using the Swedish Web-system for Enhancement and Development of Evidence-based care in Heart disease Evaluated According to Recommended Therapies registry from 1996 to 2013, the authors investigated mortality trends and the extent to which guideline-recommended interventions and treatments were used among patients undergoing HD. They reported a substantial increase in in-hospital cardiac interventions and prescription of secondary prevention medication at discharge among these patients and reported that 1-year mortality decreased substantially from 59.6% to 41.2% [18]. Recently, Lin et al. used the National Health Insurance Research Database in Taiwan to show that using more evidence-based medications for secondary prevention after myocardial infarction was associated with a lower risk of all-cause mortality in patients undergoing HD [19]. In the present study, we also found that ischemic heart disease mortality rates tended to decline from 2003 to 2021. Cardiac intervention or secondary prevention in patients undergoing HD may explain the improved survival from ischemic heart disease, but further research is needed.

This study also showed that mortality from heart failure and cardiomyopathy increased over time in patients undergoing HD. These patients have a high burden of heart failure morbidity and mortality. Standard heart failure therapies have not been proven to have similar benefits in patients with ESKD. Based on administrative data and limited echocardiographic studies, ~20% of patients undergoing HD have heart failure with reduced ejection fraction [13,20,21]. Comorbid heart failure and ESKD requiring HD have a poor prognosis [21], but few studies have examined mortality trends for heart failure in patients undergoing HD. Recent results from the large-sample China Dialysis Outcomes and Practice Patterns Study reported an association between congestive heart failure (CHF) and all-cause and cardiovascular mortalities among patients undergoing HD [22]. Among 1411 patients undergoing HD, 24.1% had a CHF diagnosis at enrollment. Overall mortality rates during the follow-up period were 21.8% and 12.0% in patients with and without CHF, respectively. CHF was associated with a 1.7-times higher risk of all-cause mortality and cardiovascular death in the study population. The JRDR data showed that the ASR for cardiac failure decreased, but the rate was attenuated in the 1998–2013 period [4]. Although recent studies of heart failure mortality in patients undergoing HD have been sparse, aging and comorbidities such as diabetes and preexisting heart failure may explain the upward trend in heart failure and cardiomyopathy mortality herein. However, further studies are needed to draw definitive conclusions.

In this study, the risk of death from infectious diseases was mixed over time but did not change from 2018 to 2021, similar to other studies [4,14]. Trends in cancer mortality in patients undergoing HD have been inconsistent, and this study showed a decrease in mortality [4,14]. Cancer survival rates in the general population in most developed countries are improving due to advances in cancer treatments and improved treatment tolerance in older adult patients with cancer [23]. However, the performance of cancer screening guidelines and newer cancer therapies is not well understood within the population of patients undergoing HD, and a tailored approach is recommended [24]. A study using the Australian and New Zealand Dialysis and Transplantation Registry 1980–2013 data demonstrated that all-site cancer mortality in those receiving HD exceeded that among the general population, but had a declining relative mortality risk [25]. While the findings herein of decreased cancer mortality cannot be attributed to recent improvements in new cancer treatments in patients undergoing HD, it does suggest the need for an individualized, risk-based approach to cancer care in high-risk populations.

This study found that the trend in crude mortality for patients undergoing HD varies with age, with a decrease seen in those aged 60 years and older. This could be explained by the decrease in the proportion of younger people starting HD over time. However, the increase in mortality in younger age groups needs further study. As shown in this study, the proportion of older and very old patients initiating HD has been increasing in recent years, and mortality rates have been decreasing over the past two decades. This may help physicians decide whether to start HD in very old patients. In this study, male and female patients had similar all-cause mortality rates, which appears to be similar to other studies that have shown reduced gender differences in survival in patients undergoing HD, as opposed to women surviving longer than men in the general population [26,27]. Fewer women than men receive HD treatment, and higher mortality rates among patients undergoing HD compared to the general population may also contribute to the reduced gender difference [3,27]. However, this does not fully explain this finding, and women may differ from men in a variety of variables, including biological factors, patient care, or factors related to society. Further research into gender differences in survival among patients undergoing HD may be warranted.

There are several limitations to this study. First, because this study is based on NHIS claim data, we could not obtain individual clinical data (e.g., primary cause of ESKD, comorbidities, and dialysis history) and dialysis-specific data (e.g., dialysis conditions, hemodialysis or hemodiafiltration, dialysis membranes, and dialysis efficiency). Second, the cause of death can be difficult to categorize in patients with ESKD, which may have led to misclassification. In patients undergoing HD, the cause of death can be multifactorial, meaning that cardiovascular and noncardiovascular diseases can overlap. Third, this study included only a South Korean patient population. Direct comparisons of mortality trends across countries are limited due to varying health policies, health insurance, and services. Nevertheless, this study included mortality data for all patients undergoing HD for nearly 20 years in South Korea allowed for a comprehensive analysis of mortality trends. Fourth, we included the COVID-19 outbreak period but cannot specify COVID-19-related mortality; despite this, we did not find that the all-cause mortality trend changed significantly from 2017 to 2021.

In conclusion, our analyses of nationally representative data showed a decrease in ASRs in patients who underwent HD from 2003 to 2021. The risk of noncardiovascular mortality decreased during the study period, while cardiovascular mortality showed an increasing trend.

## Figures and Tables

**Table 1:** Mortality rate in the study population and the general population.

	General Population							The Study Population						
	20–29	30–39	40–49	50–59	60–69	70–79	80+	20–29	30–39	40–49	50–59	60–69	70–79	80+
2003	59.6	108.4	263.8	567.7	1360.3	3688.8	11,593.4	3508.8	5231.9	7106.3	9742.5	17,661.6	24,975.9	41,678.5
2004	51.0	97.8	254.4	548.7	1315.5	3536.1	11,294.9	4038.3	4288.4	5886.8	9955.8	15,436.8	21,652.6	31,123.4
2005	54.3	96.1	239.1	511.6	1252.9	3383.7	11,053.2	4554.9	2215.9	4497.9	7945.7	13,688.4	20,749.8	31,111.1
2006	47.8	85.1	223.5	492.4	1176.5	3212.2	10,825.7	3692.3	3757.6	5600.4	9329.2	14,554.1	19,179.2	30,420.2
2007	55.1	87.1	218.4	461.5	1121.9	3086.6	10,501.5	4246.3	3993.9	6051.8	8983.7	14,285.7	18,986.4	29,926.3
2008	55.4	86.2	210.8	446.6	1052.3	2918.3	10,009.1	3104.9	3563.1	6288.5	8915.9	13,410.1	18,122.6	27,636.5
2009	56.8	92.1	206.5	433.0	1001.8	2766.0	9398.7	4100.2	3200.9	6225.9	8416.5	13,034.8	16,948.0	25,568.2
2010	54.3	87.4	201.7	420.8	971.7	2740.9	9423.9	3624.2	3686.9	6119.9	8124.6	14,184.2	17,044.5	26,034.1
2011	51.4	83.2	187.7	408.2	923.0	2623.6	9245.1	3428.6	3307.3	6593.1	8668.7	12,979.6	15,926.9	23,964.1
2012	45.0	79.5	180.5	389.9	873.5	2610.8	9466.6	4740.7	4565.9	5651.8	8299.1	13,469.2	16,400.8	23,451.1
2013	42.3	78.2	174.4	382.1	821.8	2475.5	8902.6	5223.9	4619.0	6889.5	7804.0	11,822.2	15,636.1	20,950.7
2014	39.2	75.7	165.8	372.4	781.7	2358.0	8597.5	5580.0	4442.1	6771.6	8730.8	11,713.2	15,342.1	20,223.4
2015	39.7	70.6	161.2	352.9	758.5	2333.6	8584.7	5516.3	5296.0	6853.5	8241.9	10,645.1	15,033.4	18,918.6
2016	37.4	68.8	153.6	346.9	740.8	2239.0	8393.2	4039.0	5112.8	6854.4	8508.1	10,434.8	13,978.3	19,111.2
2017	36.6	66.5	147.0	332.4	692.6	2138.0	8342.7	5483.4	6213.9	7081.9	7862.5	10,085.9	14,195.7	18,704.0
2018	37.3	69.8	148.2	330.7	684.9	2092.6	8407.7	6278.0	5889.2	7569.7	8958.1	10,417.9	14,877.1	19,029.2
2019	37.7	69.0	143.0	320.4	652.4	1948.1	7833.7	6240.7	6059.5	7868.7	9080.8	9536.9	14,263.8	18,619.7
2020	39.9	68.8	140.4	309.1	638.1	1895.5	7824.5	5555.6	7316.1	8923.0	8917.5	9393.2	14,035.6	18,049.7
2021	41.4	67.2	137.7	297.5	646.2	1873.6	7847.3	6679.0	8011.6	9375.8	9826.9	9864.7	14,690.7	18,825.1

The data represent death rates per 100,000 persons.

**Table 2:** Baseline characteristics of patients undergoing HD in Korea, 2003–2021.

	2003–2007	2008–2012	2013–2017	2018–2021
Number of patients	166,450	266,000	357,262	354,377
Men, N (%)	93,598 (56.23)	151,615 (57.00)	206,252 (57.73)	209,265 (59.05)
Age group, N (%)				
20–34 years	10,000 (6.01)	9875 (3.71)	8392 (2.35)	5911 (1.67)
35–49	35,414 (21.28)	47,945 (18.02)	49,859 (13.96)	37,879 (10.69)
50–64	61,510 (36.95)	97,839 (36.78)	12,7021 (35.55)	117,097 (33.04)
65–79	52,385 (31.47)	92,274 (34.69)	131,220 (36.73)	134,419 (37.93)
80+	7141 (4.29)	18,067 (6.79)	40,770 (11.41)	59,071 (16.67)

**Table 3:** Age-standardized mortality rates in patients undergoing HD, 2003–2021.

Year	Age-Standardized Mortality Rates (per 1000 Person-Years)		
	Total	Men	Women
2003	174.14	176.92	173.36
2004	149.61	155.14	144.06
2005	136.03	140.83	129.99
2006	139.44	143.62	134.45
2007	137.48	139.38	135.57
2008	130.63	133.16	127.84
2009	124.03	122.92	125.60
2010	126.54	127.02	126.25
2011	120.89	121.28	120.35
2012	121.34	125.82	115.34
2013	113.79	115.13	111.97
2014	114.32	116.58	111.06
2015	109.23	112.52	104.38
2016	106.99	109.74	102.92
2017	105.46	108.37	101.17
2018	111.13	113.13	107.73
2019	108.03	109.46	105.30
2020	108.00	110.71	103.43
2021	114.53	115.75	112.08

Age-adjusted mortality rates were calculated using the 2013 standard population.

**Table 4:** Annual percentage change (APC) in age-standardized mortality rates in the entire study population.

	1st Period Trend		2nd Period Trend		3rd Period Trend		4th Period Trend	
	Year	APC (95% CI)	Year	APC (95% CI)	Year	APC (95% CI)	Year	APC (95% CI)
All-cause	2003−2005	−9.8 (−12.3–−3.3) *	2005−2017	−2.3(−3.6–−1.1) *	2017−2021	1.6 (−1.2–6.5)		
Cardiovascular	2003−2013	−1.2 (−8.7–0.6)	2013−2021	3.9 (1.3–14.0) *				
Noncardiovascular	2003−2005	−10.2 (−12.7–−4.0)	2005−2017	−2.7 (−3.9–−1.7)	2017−2021	1.1 (−1.5–5.9)		
Cause specific								
Ischemic heart disease	2003−2021	−0.5 (−1.4–0.4)						
Heart failure	2003−2009	30.1 (17.7–70.7) *	2009−2012	−15.3(−26.1–8.7)	2012−2021	15.6 (7.3–50.5) *		
Cerebrovascular disease	2003−2012	−5.1 (−13.6–−2.6) *	2012−2021	2.3 (−0.4–12.2)				
Hyperkalemia/sudden death	2003−2006	−22.4 (−48.2–7.5)	2006−2009	45.9 (15.4–75.1) *	2009−2021	10.6 (−6.5–15.0)		
Infection	2003−2005	9.4 (1.9–15.5) *	2005−2014	−2.6 (−4.0–−1.8) *	2014−2018	26.9 (23.3–30.8) *	2018−2021	3.1 (−1.9–8.3)
Malignancy	2003−2007	−17.4 (−23.7–−13.3) *	2007−2021	−0.9 (−1.7–0.005)				

* *p* < 0.05.

## Data Availability

The data presented in this study are available on request from the corresponding author. The data are not publicly available due to the policy of the Korean NHIS.

## Supplementary Materials

The following supporting information can be downloaded at: https://www.mdpi.com/article/10.3390/jcm14092987/s1, Table S1: Cause of death defined by ICD-10 coding system; Table S2: ICD-10 codes for comorbidities; Table S3: Baseline characteristics of ESKD patients who start hemodialysis, 2003–2021; Table S4: Number of hemodialysis patient deaths, 2003–2021; Table S5: Annual percentage change (APC) in age-standardized mortality rates for female patients; Table S6: Annual percentage change (APC) in age-standardized mortality rates for male patients.
