# Supplementary material for: Mortality Trends in Patients Undergoing Hemodialysis, 2003–2021: Data from National Health Insurance Service in Korea
Source: J Clin Med. 2025 Apr 25;14(9):2987. doi: 10.3390/jcm14092987 (PMC12073088; doi:10.3390/jcm14092987)
Supplement: Supplementary file 1 [file jcm-14-02987-s001.zip › jcm-3570171-supplementary.pdf]

Table S1. Cause of death defined by ICD-10 coding system

Cardiovascular disease	
I26, I11.0, I13.0, I13.2, I13.9, I42, I43, I50, I20-I25, I05-09, I34-37, I44-45, I47-49, R00.0, R00.1, R00.3, R00.8, I46, E87, I60-69, G45-46	
Cause-specific mortality	
Heart failure and cardiomyopathy	I11.0, I13.0, I13.2, I13.9, I42, I43, I50
Ischemic heart disease	I20-I25
Hyperkalemia/sudden death	I46, E87
Cerebrovascular disease (transient cerebral ischemia stroke, cerebral hemorrhage)	I60-69, G45-46
Infection	A15–A19, A40–A41, B15.9, B16.1, B16.9, B17, B15.0, B16.0, B16.2, K72.0, B20–B24, A00–A09, A20-39, A42–A99, B00–B09, B25–B99, G00–G09, J10–J11, J12–J18, K65
Malignancy	C00-97

Table S2. ICD-10 codes for comorbidities

Diabetes	E10-14
Hypertension	I10-13, I15, R03.0
Heart failure	I11.0, I13.0, I13.2, I13.9, I42, I43, I50
Ischemic heart failure	I20-25
Liver cirrhosis and liver failure	K70.2, K70.3, K71.7, K74, K76.6, K76.7, I85.0, I85.9, I98.2, I98.3, K70.4, K72

Table S3. Baseline characteristics of ESKD patients who start hemodialysis, 2003 – 2021

	2003 - 2007	2008 - 2012	2013 - 2017	2018 - 2021
Number of patients	59,393	56,360	68,367	62,671
Men, N (%)	33,783 (56.88)	32,568 (57.79)	39,865 (58.31)	37,859 (60.41)
Age group, N (%)				
20 – 34 years	4132 (6.96)	2459 (4.36)	2046 (2.99)	1401 (2.24)
35 – 49	12,797 (21.55)	9274 (16.45)	8448 (12.36)	6161 (9.83)
50 – 64	20,186 (33.99)	17,971 (31.89)	20,518 (30.01)	17,178 (27.41)
65 – 79	18,815 (31.69)	20,727 (36.78)	25,774 (37.70)	23,050 (36.78)
80 +	3463 (5.83)	5929 (10.52)	11,581 (16.94)	14,881 (23.74)
Comorbidities				
Diabetes, N (%)	34,624 (58.30)	39,573 (70.21)	50,235 (73.48)	47,111 (75.17)
Hypertension, N (%)	14,222 (23.95)	15,352 (27.24)	23,041 (33.70)	24,716 (39.44)
Heart failure, N (%)	44,167 (74.36)	50,206 (89.08)	62,661 (91.65)	58709 (93.68)
Ischemic heart disease, N (%)	19,997 (33.67)	20,687 (36.71)	23,712 (34.68)	22508 (35.91)
Liver cirrhosis, N (%)	4247 (7.15)	3779 (6.71)	3783 (5.53)	3340 (5.33)

Table S4. Number of hemodialysis patient deaths, 2003-2021

	Total	2003	2004	2005	2006	2007	2008	2009	2010	2011	2012	2013	2014	2015	201
All-cause	136,302	2933	3538	4022	4899	5374	5765	6042	6210	6630	7206	7222	7804	8168	867
Cardiovascular	18,533	271	333	391	523	642	699	756	743	736	811	800	1090	1161	133

Noncardiovascular	117,769	2662	3205	3631	4376	4732	5066	5286	5467	5894	6395	6422	6714	7007	734
Cause-specific															
Ischemic heart disease	6881	93	136	142	238	280	277	292	292	287	335	329	420	478	481
Heart failure or cardiomyopathy	2015	7	6	10	13	24	47	71	62	52	54	53	84	83	134
Cerebrovascular disease	6733	153	166	209	239	293	297	302	306	296	303	297	406	405	470
Hyperkalemia/sudden death	1467	8	8	12	8	8	29	40	31	46	46	57	69	82	142
Infection	7946	67	100	137	176	159	200	217	192	217	239	252	266	378	523
Malignancy	13,587	435	507	519	476	492	485	608	632	589	704	728	768	845	857

Table S5. Annual percentage change (APC) in age-standardized mortality rates for female patients

	1st period trend		2nd period trend		3rd period trend		4th period trend	
	year	APC (95% CI)	year	APC (95% CI)	year	APC (95% CI)	year	APC (95% CI)
All-cause	2003-2005	-12.3 (-16.9 - -4.7)*	2005-2008	0.2 (-2.7 - 4.4)	2008-2016	-3.1 (-7.5 - -0.6)*	2016-2021	1.3 (-1.3 - 7.5)
Cardiovascular	2003-2009	2.0 (-1.50 - 10.6)	2009-2012	-9.0 (-12.7 - 1.8)	2012-2021	4.0 (1.6 - 8.4)*		
Noncardiovascular	2003-2005	-13.2 (-16.7 - -7.3)*	2005-2010	-1.1 (-2.3 - 3.1)	2010-2017	-3.6 (-7.4 - -2.9)*	2017-2021	2.1 (-0.5 - 7.6)
Cause specific								
Ischemic heart disease	2003-2021	-1.8 (-2.9 - -0.6)*						
Heart failure	2003-2021	11.4 (4.8 - 18.4)*						
Cerebrovascular disease	2003-2013	-3.8 (-14.4 - -1.3)*	2013-2021	2.0 (-1.6 - 16.9)				
Hyperkalemia/sudden death	2003-2006	-35.9 (-62.0 - -8.1)*	2006-2009	55.2 (16.9 - 94.1)*	2009-2021	9.0 (-8.8 - 13.1)		
Infection	2003-2013	1.2 (-6.3 - 4.5)	2013-2021	15.3 (10.1 - 30.0)*				
Malignancy	2003-2006	-24.2 (-37.0 - -14.5)*	2006-2021	-1.4 (-2.5 - 0.1)				

*P < 0.05

Table S6. Annual percentage change (APC) in age-standardized mortality rates for male patients

	1st period trend		2nd period trend		3rd period trend		4th period trend	
	year	APC (95% CI)	year	APC (95% CI)	year	APC (95% CI)	year	APC (95% CI)
All-cause	2003-2005	-9.7 (-12.3 - -3.5)*	2005-2016	-2.4 (-3.7 - -1.2)*	2016-2021	0.7 (-1.2 - 5.4)		
Cardiovascular	2003-2011	-2.0 (-8.6 - 0.2)	2011-2021	3.7 (1.9 - 8.2)*				
Noncardiovascular	2003-2005	-9.9 (-12.3 - -5.3)*	2005-2017	-2.8 (-3.4 - -2.2)*	2017-2021	1.0 (-1.0 - 5.3)		
Cause specific								
Ischemic heart disease	2003-2021	0.2 (-1.1 - 1.5)						
Heart failure	2003-2021	11.8 (9.1 - 14.5)*						
Cerebrovascular disease	2003-2011	-6.6 (-15.2 - -3.6)*	2011-2021	2.3 (-0.03 – 8.8)				
Hyperkalemia/sudden death	2003-2021	14.2 (11.3 – 17.3)*						
Infection	2003-2014	-3.1 (-5.7 - -1.1)*	2014-2018	26.6 (-1.0 – 40.3)	2018-2021	2.8 (-12.0 – 15.8)		
Malignancy	2003-2007	-17.0 (-24.7 - -12.3)*	2007-2021	-1.0 (-2.0 - 0.05)				

*P < 0.05
